# Bioelectrochemically triggered apoferritin-based bionanoreactors: synthesis of CdSe nanoparticles and monitoring with leaky waveguides†

**DOI:** 10.1039/d3na01046e

**Published:** 2023-12-14

**Authors:** Angelo Tricase, Bushra Alhenaki, Verdiana Marchianò, Luisa Torsi, Ruchi Gupta, Paolo Bollella

**Affiliations:** a Department of Chemistry, University of Bari Aldo Moro Via E. Orabona, 4 70125 Bari Italy paolo.bollella@uniba.it; b Centre for Colloid and Surface Science, University of Bari Aldo Moro Via E. Orabona, 4 70125 Bari Italy; c School of Chemistry, University of Birmingham Birmingham B15 2TT UK r.gupta.3@bham.ac.uk

## Abstract

Herein, we describe a novel method for producing cadmium-selenide nanoparticles (CdSe NPs) with controlled size using apoferritin as a bionanoreactor triggered by local pH change at the electrode/solution interface. Apoferritin is known for its reversible self-assembly at alkaline pH. The pH change is induced electrochemically by reducing O_2_ through the application of sufficiently negative voltages and bioelectrochemically through O_2_ reduction catalyzed by laccase, co-immobilized with apoferritin on the electrode surface. Specifically, a Ti electrode is modified with (3-aminopropyl)triethoxysilane, followed by glutaraldehyde cross-linking (1.5% v/v in H_2_O) of apoferritin (as the bionanoreactor) and laccase (as the local pH change triggering system). This proposed platform offers a universal approach for controlling the synthesis of semiconductor NPs within a bionanoreactor solely driven by (bio)electrochemical inputs. The CdSe NPs obtained through different synthetic approaches, namely electrochemical and bioelectrochemical, were characterized spectroscopically (UV-Vis, Raman, XRD) and morphologically (TEM). Finally, we conducted online monitoring of CdSe NPs formation within the apoferritin core by integrating the electrochemical system with LWs. The quantity of CdSe NPs produced through bioelectrochemical means was determined to be 2.08 ± 0.12 mg after 90 minutes of voltage application in the presence of O_2_. TEM measurements revealed that the bioelectrochemically synthesized CdSe NPs have a diameter of 4 ± 1 nm, accounting for 85% of the size distribution, a result corroborated by XRD data. Further research is needed to explore the synthesis of nanoparticles using different biological nanoreactors, as the process can be challenging due to the elevated buffer capacitance of biological media.

## Abstract 2

Enzymatic processes, specifically laccase (Lac) catalysis, control molecular or nanomaterial synthesis within apoferritin by inducing a local pH change. Leaky waveguides technique is used for real-time monitoring of CdSe nanoparticles synthesis.

## Graphical abstract

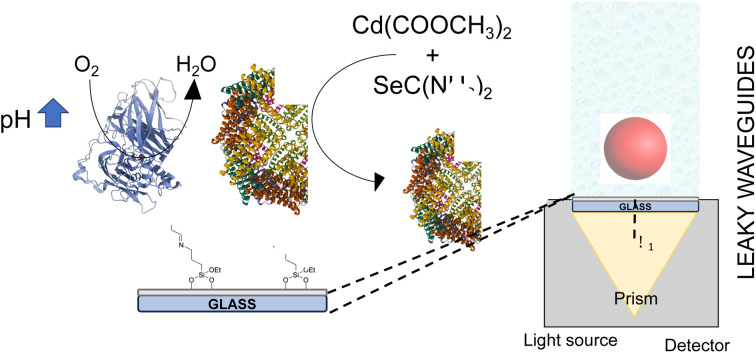

## 1. Introduction

Self-assembling proteins have emerged as an innovative approach for developing programmable nanomaterials.^1,2^ This approach utilizes proteins and peptides as intelligent building blocks and supramolecular templates, enabling the design of bioinspired nanomaterials with improved solubility, biocompatibility, and reduced toxicity.^3,4^ These materials also exhibit desirable characteristics such as biodegradability, stability, ease of surface modification and bioconjugation, and precise control over particle size, enabling targeted drug delivery and other applications.^5^ Additionally, these biomimetic materials serve as nanocarriers, providing a highly efficient framework for encapsulating and controlling the release of various cargoes including nanoparticles, enzymes, and drugs, while also enabling site-specific targeted delivery.^6,7^ Furthermore, they offer nanoscale interior compartments, acting as nanoreactors for synthesizing and incorporating nanomaterials with adjustable physicochemical properties.^8^

Ferritin, a protein nanocage that self-assembles, has a natural function in the storage of iron and plays a vital role in regulating iron metabolism and maintaining iron homeostasis.^9,10^ Prior research has demonstrated that ferritin, an iron storage protein that self-assembles into an 8 nm polypeptide cage, can effectively facilitate the synthesis and confinement of various inorganic materials, including uranyl oxide,^11^ manganese oxide,^12^ and magnetite.^13^ The native iron oxide core can undergo *in situ* sulphidation, or the empty apo-protein can be reconstituted with iron and then sulphidated to generate the corresponding sulphide.^14^ In each instance, the nanoparticles are enclosed within the protein cavity, limiting their growth to the internal dimension and resulting in dispersed solutions with potential biocompatibility and bioactive properties.^15^ Research conducted by Pead *et al.* has revealed that the apo-protein possesses the ability to bind with other metal ion species, opening up the possibility of producing non-native bio-inorganic nanomaterials.^16^ Douglas *et al.* demonstrated this capability by synthesizing cobalt oxyhydroxide.^17^ The protein's capacity to uptake non-native metal ions depends on the accessibility of binding sites through diffusion within the molecular channels inherent in the polypeptide cage. Hence, it can be employed as nanocavity for the controlled synthesis of semiconductors nanoparticles (*e.g.*, CdSe NPs) regulating the uptake of other metals (excluding iron) and small organic molecules required in the biochemical process.^18^

Controlled synthesis of biomolecules or bionanomaterials, stimulated by signals, play a key role in several research areas, spanning from *in vitro* biotechnological studies to *in vivo* biomedical applications.^19,20^ Over the past few decades, there has been an extensive exploration of synthetic mechanisms triggered by various physical or chemical stimuli, including light, magnetic field, temperature, electrochemical redox processes, mechanical stress, pH gradient, and the addition of different molecular or biomolecular species.^21,22^ Among these, electrochemically stimulated synthesis has garnered significant attention due to its simplicity and versatility. Researchers have employed electrochemical methods such as disassembling polymeric matrices, cleaving chemical bonds through redox reactions, or exploiting electrostatic attraction/repulsion of charged molecules to achieve controlled synthesis of molecules or nano-sized species.^23,24^ Combining electrochemical processes with local pH gradient has led to the development of intriguing systems for signal-regulated biomolecular synthesis. In these systems, electrochemical reduction of O_2_ generates a local increase in pH due to the consumption of H^+^ ions required for the formation of H_2_O.^25^ The process relies on oxygen, a readily available chemical. However, one limitation of this method is that reducing oxygen requires relatively high negative potentials, typically at least −0.6 V *vs.* Ag/AgCl in an aqueous solution with neutral pH, using a titanium (Ti) electrode (other electrodes may require even higher potentials). Although this potential is manageable in controlled environments using modified releasing electrodes, it can lead to undesired side electrochemical reactions in complex biological fluids or potential future biomedical applications. To overcome this obstacle, catalysing the reduction of oxygen can lower the necessary potential for the process.^26,27^

Recently, leaky waveguides (LWs) have been proposed as label-free optical sensors.^28^ LWs are based on low-refractive index materials like hydrogels. Light within the LWs is constrained by factors beyond total internal reflection (TIR) at one or both interfaces. A basic form of such a device involves a hydrogel layer situated between a substrate with a higher refractive index and a cover layer with a lower refractive index, forming a slab waveguide. In this structure, TIR occurs at the waveguide–cover layer interface, while Fresnel reflection takes place at the waveguide–substrate interface. Due to the leakiness of the substrate–waveguide interface, light can be coupled into and out of the waveguide through the substrate using a prism. These structures have been referred to by other researchers as hydrogel optical waveguides (HOWs) due to the material utilized in constructing the waveguide. LWs phenomena have been demonstrated across a broad spectrum of wavelengths, spanning from short wavelengths limited by material absorption or scattering losses to long wavelengths constrained by waveguide cutoff. The apparatus is functional within the range of 320 nm (restricted by absorption in the BK7 substrate, used as prism material) to 950 nm (limited by cutoff). Hence, LWs enable the determination of the optical dispersion of sample species by assessing the resonance angle in relation to wavelength across an extensive spectrum. This method has been recently implemented to monitor the variation of the refractive index related to the accumulation of iron atoms within ferritin core.^29^

In this paper, we report a size-controlled synthesis of cadmium-selenide (CdSe) nanoparticles (NPs) using apoferritin as bionanoreactor. Apoferritin is well-known to self-assemble reversibly at alkaline pH. In this regard, CdSe NPs synthesis was triggered through apoferritin self-assembling upon local pH increase at the interface electrode/solution. Local pH change was triggered electrochemically through O_2_ reduction by applying sufficiently negative voltages, and bioelectrochemically through O_2_ reduction catalysed by laccase (co-immobilized onto the electrode surface with apoferritin). In particular, a Ti electrode was modified with (3-aminopropyl)triethoxysilane followed by glutaraldehyde cross-linking (1.5% v/v in H_2_O) of apoferritin (as bionanoreactor) and laccase (as local pH change triggering system). The proposed platform provides a universal approach to control semiconductors NPs synthesis within a bionanoreactor exclusively governed by (bio)electrochemical inputs. The CdSe NPs obtained through different synthetic approaches, namely electrochemical and bioelectrochemical, were spectroscopically (UV-Vis, Raman, XRD) and morphologically (TEM) characterised. Finally, we performed the online monitoring of CdSe NPs formation within apoferritin core by integrating the electrochemical system with LWs.

## 2. Results

**Fig. 1:**
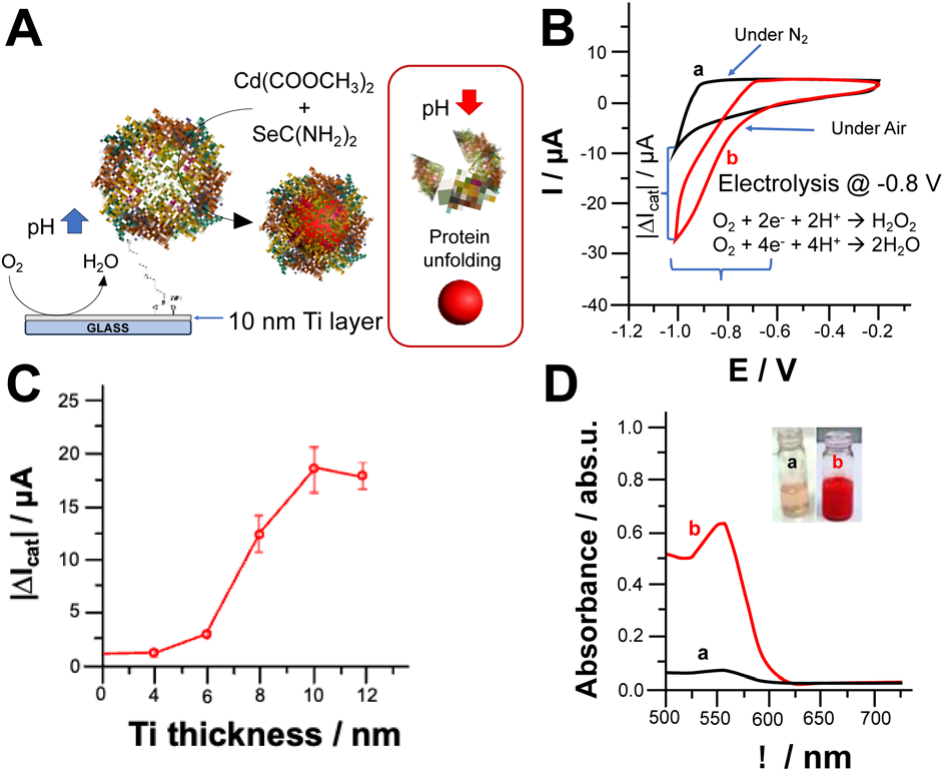
(A) Scheme of electrochemically triggered local pH change; (B) cyclic voltammograms recorded with the Ti modified electrode with ferritin (1 cm^2^ geometrical area) with the absence (a) and presence of O_2_ (in equilibrium with air). Potential scan rate, 5 mV s^−1^. Background electrolyte: NH_3_/acetate buffer (3 mM, pH 6.5 containing 0.1 M Na_2_SO_4_); (C) catalytic current measurements at −0.8 V *vs.* Ag/AgCl correlated to Ti thickness; (D) UV-Vis spectra of reaction media after applying −0.8 V *vs.* Ag/AgCl for 30 min in the absence (a) and presence of O_2_ (in equilibrium with air) – insets: photo of reaction media before (a) and after (b) electrochemically triggered CdSe NPs synthesis.

We investigated the oxygen reduction reaction (ORR) on Ti electrodes modified with apoferritin as schematically reported in Fig. 1A. The cyclic voltammograms shown in Fig. 1B depict the electrochemical behaviour of the electrode in the absence (a) and presence (b) of oxygen (O_2_). It is important to note that both electrochemical processes occurred at potentials more negative than −0.6 V *vs.* Ag/AgCl. The effect of Ti layer deposited onto glass slides on electrochemical ORR has been investigated. Fig. 1C report the catalytic current measured at −0.8 V. Ti thickness, where the current output rises to 18.4 ± 2.6 μA (1 cm^2^ as geometric area) achieving a plateau. This might be ascribable to the low roughness of metal layer deposited onto the glass.^30^ The reduction of O_2_ can lead to the formation of either hydrogen peroxide (H_2_O_2_) or water (H_2_O) depending on the properties of the electrode interface and the applied potential.^27^ The pH value at the steady state, near the surface of the electrode, is influenced by the rates of two key processes: the consumption of hydrogen ions during the electrochemical reduction of O_2_, and the diffusion of hydrogen ions from the bulk solution to the electrode surface, driven by the concentration gradient of H^+^ ions.^23,25^ To assess the pH gradient at the electrode surface, we used a pH sensitive fluorescent dye, namely 3,4′-dihydroxy-3′,5′-bis-(dimethylaminomethyl)flavone (FAM345), imaging the pH gradient at the electrode surface by using confocal fluorescence microscopy (CFM). After applying −0.8 V over 600 s, we could observe a thickness of 144 ± 11 μm (data not shown) and a pH gradient of 3.5 pH units based on the calibration curve performed by using CFM (Fig. S1 and S2†).

Fig. 1D shows the UV-Vis spectra of reaction solution containing the metal precursor, namely cadmium acetate, and selenourea which form CdSe nanoparticles upon local pH shift with the application of −0.8 V for 30 minutes in the absence and presence of O_2_ (curves a and b respectively). In the absence of O_2_, the solution turns pale orange because of selenourea photodegradation, while in the presence of O_2_, the solution turns red with the appearance of a UV-Vis peak at 552 nm, which agrees with the results reported in the literature.^31^ Several experiments also showed a blue shift at the edges of UV-Vis spectra mainly related to the quantum confinement.^31^

The electrochemically stimulated CdSe NPs synthesis was monitored by measuring UV-Vis spectra in bulk solution, Fig. S3.† Prior voltage application 30 minutes, the electrode was soaked in the reaction solution to measure uneven side reactions (*e.g.*, photodegradation of selenourea). Afterwards, −0.8 V *vs.* Ag/AgCl was applied under N_2_ for 30 min to prove that CdSe NPs could be logically triggered only in the presence of O_2_ and applying the ORR potential.

**Fig. 2:**
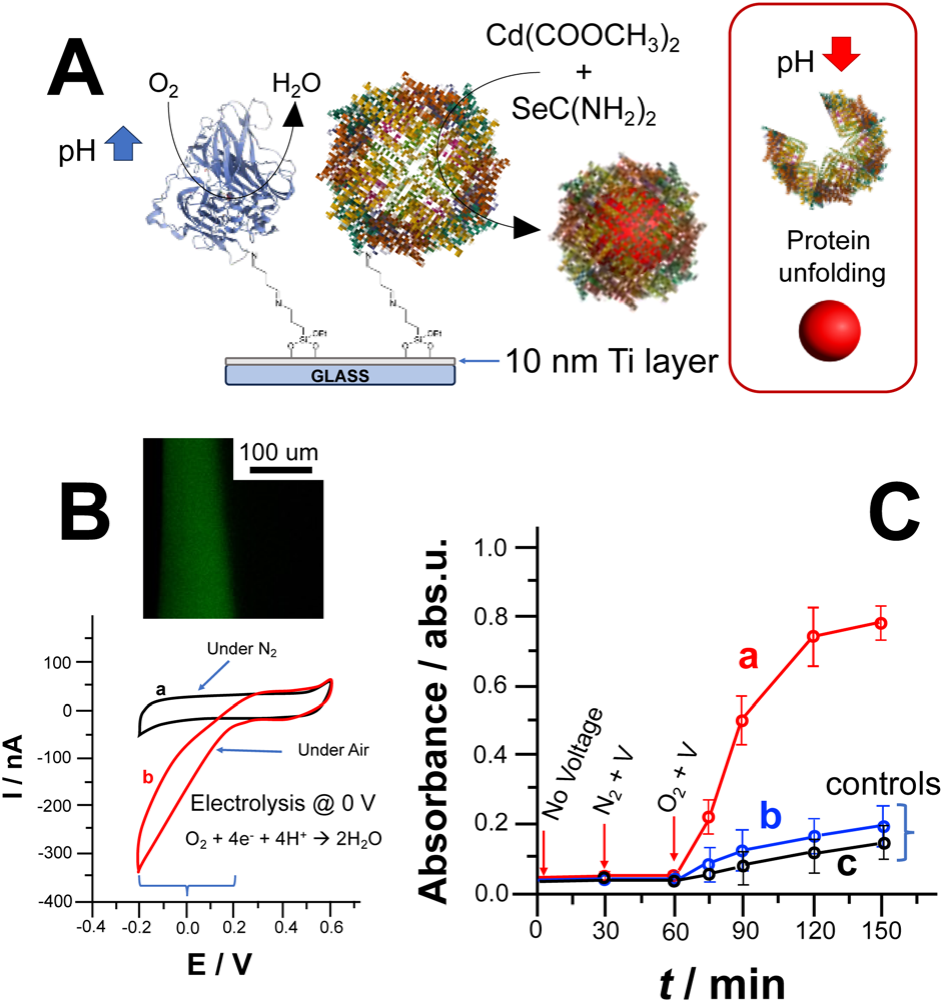
(A) Scheme reporting Ti electrode modified with laccase/apoferritin, notably as pH shift trigger and bionanoreactor, and CdSe NPs synthesis upon pH shift due to ORR followed by particles release; (B) cyclic voltammograms recorded with the TiO_2_ modified electrode with ferritin and laccase (1 cm^2^ geometrical area) with the absence (a) and presence of O_2_ (in equilibrium with air). Potential scan rate, 5 mV s^−1^. Background electrolyte: NH_3_/acetate buffer (3 mM, pH 6.5 containing 0.1 M Na_2_SO_4_) – inset: CFM image of pH gradient at the electrode surface; (C) UV-Vis kinetics measurements of reaction media after applying 0 V *vs.* Ag/AgCl for (a) CdSe NPs synthesis upon local pH change induced by ORR catalysed by laccase, (b) control experiment for CdSe NPs synthesis without laccase performed applying 0 V *vs.* Ag/AgCl and (c) control experiment without applying any voltage.

CdSe NPs formation could also be triggered by lowering the ORR reduction potential by co-immobilizing laccase (Lac) and apoferritin. Notably, laccase belongs to the multicopper oxidases (MCOs) protein family enabling ORR at a potential closer to the thermodynamic potential of O_2_/H_2_O reaction occurring at 0.69 V *vs.* Ag/AgCl (pH 7). Fig. 2A depicts the covalent co-immobilization of laccase and apoferritin onto Ti electrodes. Apoferritin hosts the NPs reaction with the uptake of synthesis precursors within its core.^3,25^ Thus, we immobilised laccase and apoferritin on Ti electrodes as shown in Fig. 2A.

The modified Ti electrode was characterized by recording CVs in the absence (curve a) and presence (curve b) of oxygen (O_2_), as shown in Fig. 2B. In addition, the pH gradient thickness was measured by exploiting CFM to prove the effectiveness of ORR to trigger CdSe NPs synthesis (Fig. 2B inset).^26,32^ After applying 0 V over 600 s, we could observe a thickness of 85 ± 9 μm and a pH gradient of 3 pH units.

The bioelectrochemically stimulated CdSe NPs synthesis was monitored by measuring UV-Vis spectra of the bulk solution, Fig. 2C, curve (a). Prior to voltage application for 30 minutes, the electrode was soaked in the reaction solution to measure uneven side reactions (*e.g.*, photodegradation of selenourea).^18^ Afterwards, 0 V *vs.* Ag/AgCl was applied under N_2_ for 30 min to show that CdSe NPs could be triggered only in the presence of O_2_ and applying the ORR potential. Considering the calibration curve for CdSe NPs (see ESI†), the amount of CdSe NPs bioelectrochemically produced is 2.08 ± 0.12 mg after 90 min (voltage application in presence of O_2_, quantification based on calibration curve reported in Fig. S3A and B†). Fig. 2C, curves (b) and (c), shows control experiments performed in the absence of laccase (triggering ORR at lower potential) and without applying potential at fully modified electrode, respectively. In both cases, there is a slight absorbance increase mainly due to photodegradation of unreacted selenourea.

**Fig. 3:**
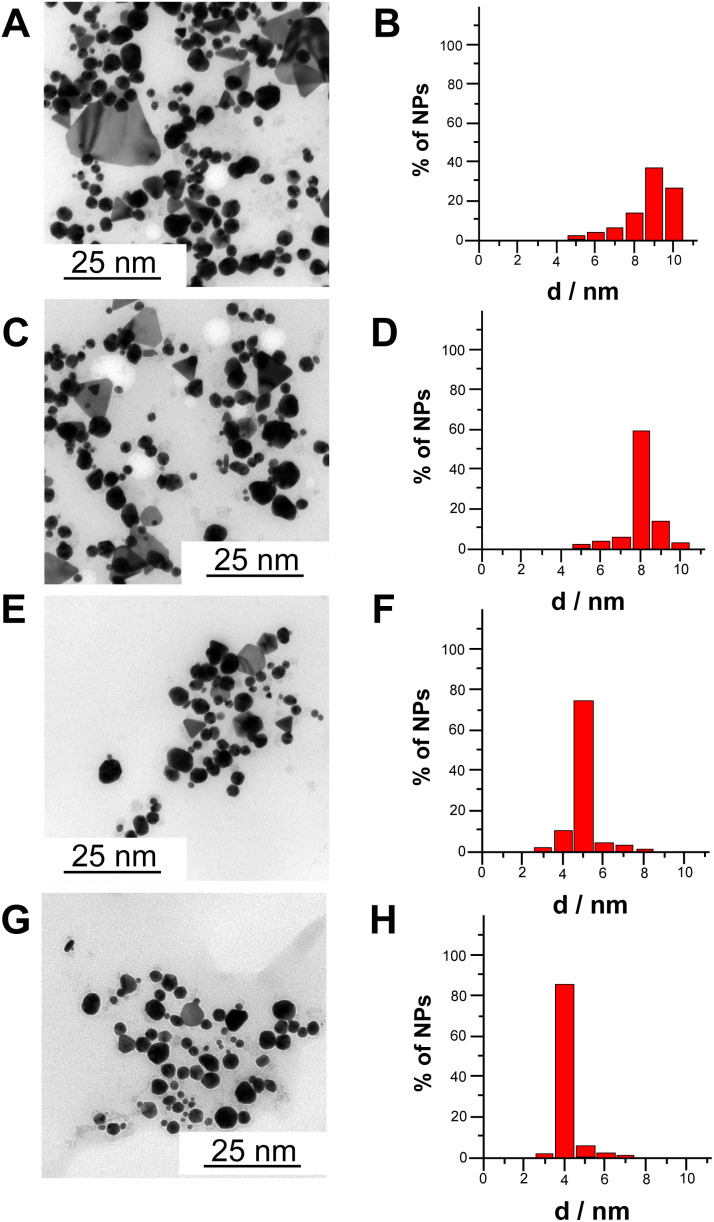
TEM pictures and corresponding size distribution for CdSe NPs synthetized by chemical synthesis without apoferritin (A and B), chemical synthesis with apoferritin (C and D), electrochemical synthesis with apoferritin (E and F) and bioelectrochemical synthesis with apoferritin (G and H).

Fig. 3A–H shows transmission electron microscopy (TEM) images and extracted size distribution of CdSe NPs synthesized according to four different methods, namely chemical synthesis without apoferritin (A and B), chemical synthesis with apoferritin (C and D), electrochemical synthesis with apoferritin (E and F) and bioelectrochemical synthesis (G and H). Notably, the average diameters of CdSe NPs are 9 ± 2 nm (40% of size distribution), 8 ± 2 nm (60% of size distribution), 5 ± 1 nm (75% of size distribution) and 4 ± 1 nm (85% of size distribution), chemically synthesized without apoferritin, chemically synthesized with apoferritin, electrochemically synthesized with apoferritin and bioelectrochemically synthesized with apoferritin, respectively. The progressive decrease in nanoparticles size and the increasing percentage of monodistribution can be ascribed to the presence of apoferritin as capping agent (protein core of 8 nm) and to local pH change ensuring a gradual apoferritin self-assembling without protein denaturation.

**Fig. 4:**
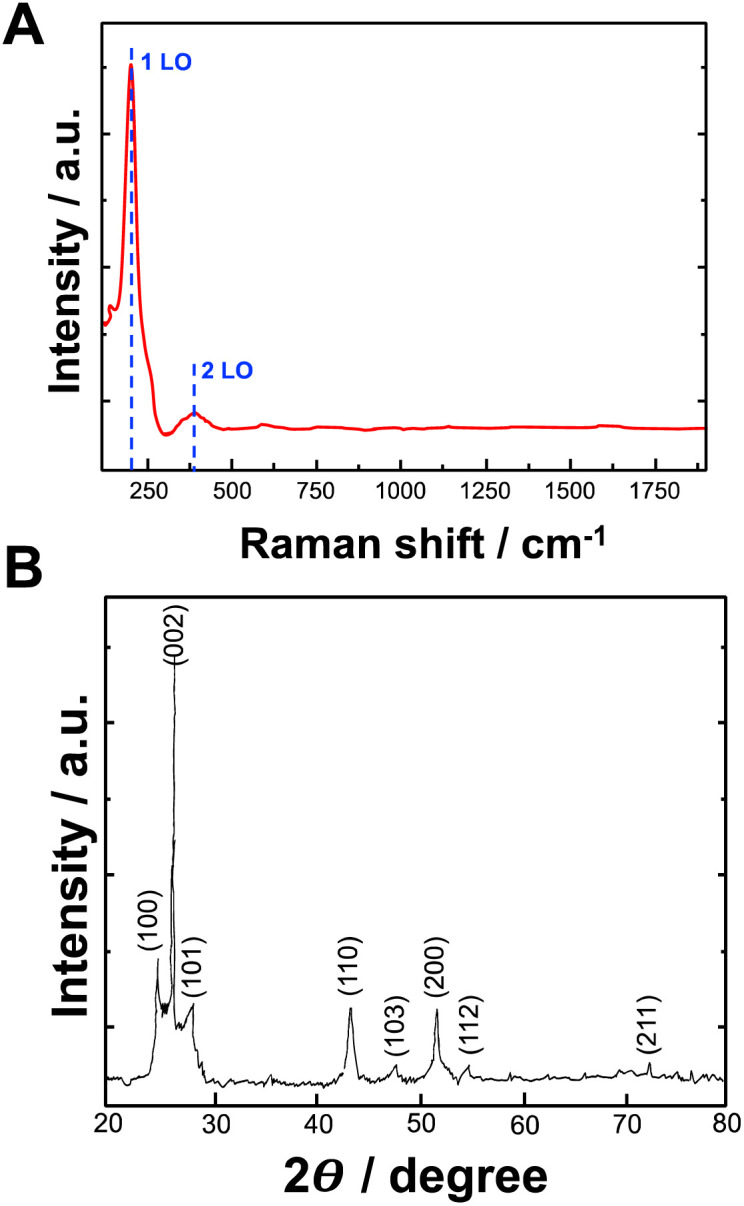
(A) Raman spectrum of CdSe NPs synthesized by using bioelectrochemical approach; (B) X-ray diffraction pattern of CdSe NPs synthesized by using bioelectrochemical approach.

Raman spectroscopy was used to determine the formation of bioelectrochemically produced CdSe. Fig. 4A shows the Raman spectrum of CdSe. The Raman longitudinal optical (LO) vibration peaks of CdSe were located at 206 cm^−1^ and 380 cm^−1^, representing 1LO and 2LO peaks, respectively.

The investigation of the crystal structure and crystallite size of the produced nanoparticles involved the generation of diffraction patterns, XRD, from crystalline powder samples at room temperature. In Fig. 4B, the XRD pattern illustrates the formation of CdSe NPs synthesized with bioelectrochemically induced local pH change. The peaks observed in the XRD patterns at 2*θ* values of 25.1, 26.5, 28.2, 43.8, 47.9, 51.9, 54.6, and 72.4° closely correspond to the (100), (002), (101), (110), (103), (200), (112), and (211) crystalline planes of an hexagonal CdSe structure (data compared with the International Centre for Diffraction Data (ICDD) PDF 02-0330), respectively.

The mean crystalline size can be determined from the XRD peak width using the Debye–Scherrer equation (eqn (1)),^33^ expressed as:1
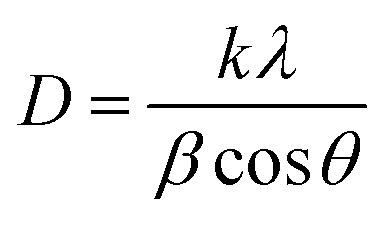
Here, *k* is a constant with a value of 0.9, *λ* represents the wavelength of the X-ray radiation (0.1542 nm), *θ* is the diffraction angle, and *β* is the full width half maximum (FWHM). By applying the Debye–Scherrer equation to the (002) reflection of the XRD pattern, the average crystalline size of CdSe nanoparticles was determined. The calculation yielded an average crystalline size of 4.2 ± 0.9 nm.

**Fig. 5:**
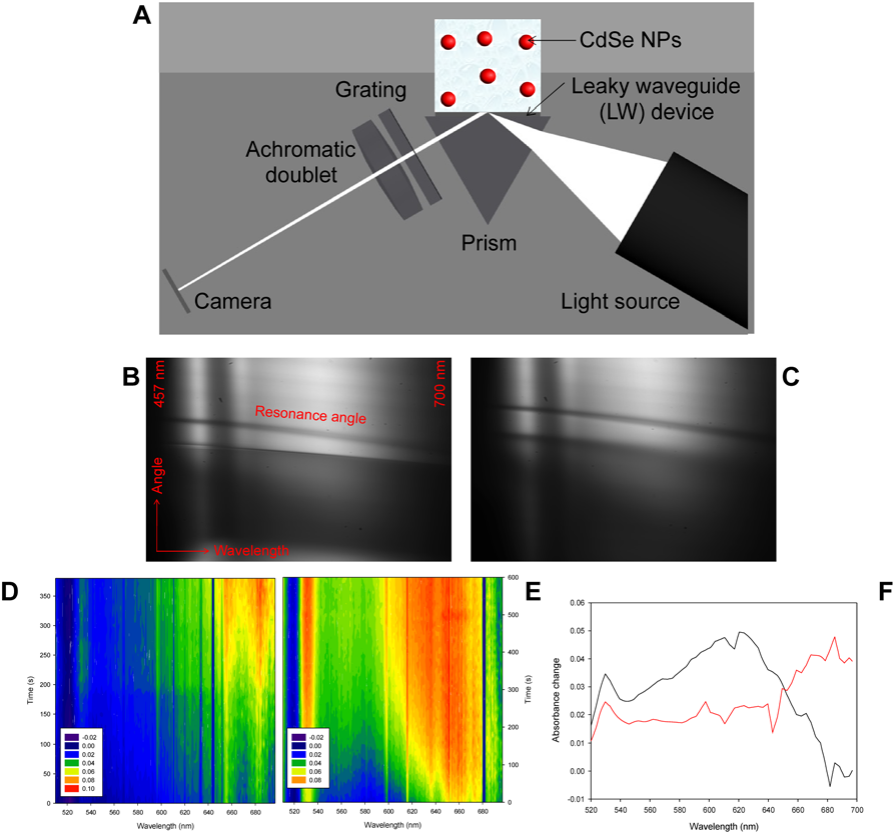
(A) Scheme of leaky waveguides (LWs) for monitoring; 2D reflectivity curves of a LW with chemically formed NP suspension at time (B) 0 s and (C) 300 s; absorbance *versus* wavelength and time obtained using LW spectroscopy for (D) chemically and (E) bioelectrochemically synthesised NPs; (F) absorbance change measured using LW spectroscopy from 0 to 300 s for chemically (red curve) and from 0 to 600 s for bioelectrochemically (black curve) formed NPs.

Fig. 5A shows a schematic of leaky waveguide (LW) spectroscopy instrumentation for studying CdSe NPs. This aims at providing a novel hybrid system as a proof-of-concept combining the newly designed bioelectrochemically triggered reaction with an optical method, which has previously been used to monitor the average content of metal precursor encapsulated by ferritin and apoferritin.^28,29^

NPs in de-ionised water were dispensed on LW and 2D reflectivity curves were captured for 600 s at a temporal resolution of ∼10 s. Exemplar reflectivity curves obtained at 0 and 300 s for a LW with chemically formed NP suspension on top are shown in Fig. 5B and C. Because of the high refractive index (∼2.64)^34^ and strong optical absorption of the CdSe NPs, the resonance curve shows a shift to higher angles and a decrease in intensity at resonance angle at all wavelengths as the NPs diffuse to the LW. Reflectivity curves of LWs were analyed using the procedure described above to obtain absorbance *versus* time and wavelength contour plots shown in Fig. 5D and E. Fig. 5F shows that there is a significant difference in the peak wavelength and time taken to diffuse to the LW for chemically and bioelectrochemically formed NPs. The chemically formed NPs absorb at longer wavelength and diffuse to the LW faster than the bioelectrochemically formed NPs, which may be because the chemically formed NPs are larger on average than the bioelectrochemically formed NPs. Fig. 5F shows the absorbance change between 0 and 300 s for the chemically formed NPs and between 0 and 600 s for the bioelectrochemically formed NPs, showing the shorter absorbance peak wavelength of the bioelectrochemically formed NPs.

## 3. Conclusions

This proof-of-concept study demonstrated the possibility to establish an electrochemical and bioelectrochemical control over a NPs synthetic process stimulated by local pH shift. Furthermore, the possibility to apply a novel optical method based on leaky waveguides has been proved to quantitatively monitor CdSe NPs. Considering the universal role of apoferritin as bionanoreactor, this approach can be extended to a plethora of NPs synthetic processes triggered by local pH change.^35^ The pH gradient induced at the electrode surface was 3.5 (thickness: 144 ± 11 μm) and 3 units (thickness: 85 ± 9 μm) by using electrochemical and bioelectrochemical inputs. The amount of CdSe NPs bioelectrochemically produced resulted to be 2.08 ± 0.12 mg after 90 min (voltage application in presence of O_2_). TEM measurements showed that the CdSe NPs bioelectrochemically synthesized exhibit a diameter of 4 ± 1 nm (85% of size distribution), further confirmed by XRD data. Additional investigations are required for synthesizing nanoparticles using various biological nanoreactors because the process can be complicated by the high buffer capacitance of biological media.

## 4. Methods

### 4.1 Chemicals and materials

All the commercial reagents: cadmium acetate (Cd(CH_3_COO)_2_), selenourea (SeC(NH_2_)_2_), ammonium hydroxide 30% v/v (NH_4_OH), sodium acetate (CH_3_COONa), sodium sulfate (Na_2_SO_4_), (3-aminopropyl)triethoxysilane (APTES), (4-(2-hydroxyethyl)-1-piperazineethanesulfonic acid) (HEPES), glutaraldehyde grade II, cadmium selenide (pristine CdSe, −325 mesh particle size, 99.99% trace metals basis, electronic grade), glutaraldehyde 25% in H_2_O, Apoferritin from Equine Spleen (CAS 9013-31-4, is a native protein shell of ferritin molecule lacking iron), ethanol, and 0.1 M acetic acid were purchased by Merck Millipore (formerly Sigma Aldrich). Chitosan (molecular weight 100–300 kDa and 90% deacetylated) and Decon 90 were purchased from Fisher (Loughborough, U.K.). Standard microscope glass slides of 1 mm thickness were purchased from VWR (Leicestershire, U.K.).

Apoferritin was solubilized in 10 mM HEPES buffer pH 7.2 at concentration of 0.5 mg mL^−1^. Laccase (Lac) was kindly provided by Amano Enzyme. Lac (concentration: 3.6 mg mL^−1^, activity 300 U mL^−1^), dissolved in 20 mM acetate buffer pH 6 containing Na_2_SO_4_ (stored in aliquots at −20 °C).

A pH-sensitive fluorescent dye, 3,4′-dihydroxy-3′,5′-bis-(dimethylaminomethyl)flavone (FAM345), was synthesized based on a previously reported procedure. A solution of 0.254 g (1 mmol) of 3,4′-dixydroxyflavone, and 0.404 g or 0.54 mL (4 mmol) of *N*,*N*,*N*′,*N*′-tetramethyldiaminomethane in 5 mL of dry dioxane was boiled 4–5 h, until a yellow–orange precipitate was formed. It was filtered off and washed with dioxane.^36^

All solutions were prepared using Milli-Q water (18.2 MΩ cm, Millipore, Bedford, MA, USA).

### 4.2 Instrumentation

Cyclic voltammetry and amperometry experiments were performed using a PalmSens4 electrochemical workstation equipped with PSTrace 5.6v software. A BASi Ag|AgCl|KCl, 3 M (all potential values reported in the paper need to be considered toward this reference) and a platinum wire were used as reference and counter electrodes, respectively. A Ti electrode, which was prepared by e-beam evaporation of titanium on glass, (geometric area = 1 cm^2^) was used as a working electrode.

Confocal fluorescent microscopy was employed to observe the local pH gradient layer induced at the electrode surface. FAM345 was detected at 470–600 nm exciting at 405 nm. A calibration curve was performed by recording images of the pH sensitive dye in solutions at different pH values.^26^

Absorbance of NPs was measured using a Shimadzu UV-2450 UV-Vis spectrophotometer with 1 mL (10 mm optical path) poly(methyl methacrylate) (PMMA) cuvettes. NPs were also characterised by measuring micro-probe Raman back-scattering excited at 532 nm wavelength laser using a NT-MDT NTEGRA system. A 50× microscope objective was used to focus the incident laser beams to a spot with a diameter of ∼1 μm. XRD patterns were recorded on a Shimadzu X-ray diffractometer XRD-3A with Cu-Kα radiation (Shimadzu model 6000, Lelyweg1, Almelo, The Netherlands), *λ* = 0.15418 nm. Transmission electron microscopy (TEM) experiments were performed with a Hitachi HF-3300 ETEM – environmental transmission electron microscope equipped with an Oxford EDX module.

Equally, NPs were characterised using leaky waveguide (LW) spectroscopy. As shown in Fig. 5A, the LW instrumentation comprised of a BK7 equilateral prism (Qioptic Photonics). A thin layer of refractive index matching oil was sandwiched between the top of the prism and the glass substrate of a LW device. Detailed information on fabrication of LW devices is provided in the ESI.† The LW device was illuminated with a wedge-shaped white light beam. The white light source was formed by combining the outputs of two LEDs (LXZ2-5070, RS Components and SMB1N-490H-02, Rothner Lasertechnik) using a multimode fiber optic coupler (TM200R5S1A, Thorlabs). The output of the optical fiber was expanded to a diameter of 25 mm and then passed through a cylindrical lens (40 YD 25, Comar Optics). The light reflected from the LW device was passed through a transmission grating (Thorlabs GT25-03, 300 lines mm^−1^, blaze angle 17.5°) to disperse it and then an achromatic doublet to focus it onto the camera (MER-2000-19U3M-L, Daheng Imaging). A slit was used before the grating to ensure that only light which passed through the flow cell region of the device was dispersed and focused onto the camera.

Fluids were pumped through the flow cell mounted on top of LW using a peristaltic pump (Minipuls 3, Gilson, Bedfordshire, U.K.) at a flow rate of 0.2 mL min^−1^. The flow cell was made by CNC machining. The flow cell was made of 3 mm thick black PMMA and had a recessed cavity with a circular channel of radius 4 mm wide and depth 0.2 mm. The channel was surrounded by a groove 1 mm wide and 0.75 mm deep for mounting an O-ring. The flow cell was placed on the LW and held in place using a fixture.

The details of the procedure used to analyse 2D reflectivity curves of LWs, which in turn provided the absorption spectra of NPs, is provided in the ESI.†

### 4.3 Synthesis of CdSe nanoparticles (NPs)

#### 4.3.1. Chemical synthesis of CdSe NPs without apoferrtin

Cd^2+^ was stabilized by ammonia solution in order to form positively charged tetraaminecadmium ions (Cd(NH_3_)_4_^2+^). Se^2−^ was designed to be supplied from selenourea. Selenourea is comparatively unstable in an aqueous solution and slowly degrades to release Se^2−^ into the reaction solution. The reaction mixture solution of 1 mM cadmium acetate and 5 mM selenourea was prepared adjusting the pH to 8 by adding 2.5 mL of ammonia solution. The solution changed colour from thin yellow to clear red in 5 minutes, providing a visual confirmation of the formation of NPs. The reaction mixture was left for 3 h and all processes were carried out at room temperature. After 3 h, the reaction mixture was centrifuged (15 000 rpm for 20 minutes) to remove the precursors and exchange it with HEPES buffer (10 mM, pH 7.2).

#### 4.3.2. Chemical synthesis of CdSe NPs with apoferrtin

The reaction mixture containing Cd^2+^ and Se^2−^ in ammonia solution at required pH was prepared as above. To this reaction mixture solution, apoferritin molecules were added with the final concentration of 0.5 mg mL^−1^. In the presence of apoferrtin, the solution changed colour from thin yellow to clear red gradually. The reaction mixture was left 3 h and all processes were carried out at room temperature. There was little precipitation, indicating that CdSe NPs were synthesized in the apoferritin cavity and the protein shells made them dispersed. This core–shell structure makes it easy to handle NPs in an aqueous solution.

#### 4.3.3. Electrochemical synthesis of CdSe NPs with apoferritin

The reaction mixture solution of 1 mM cadmium acetate and 5 mM selenourea was prepared in NH_3_/acetate buffer (3 mM, pH 6.5). The pH was changed by applying −0.8 V *vs.* Ag/AgCl, which caused oxygen reduction reaction (ORR) occurring at the electrode surface (potential applied for 90 minutes).

CdSe NPs can be synthesized in the absence of apoferritin but their size will not be controlled, resulting in NPs with high dispersion distribution in terms of size. To control the size of NPs, apoferritin was immobilized on the surface of titanium electrode. The Ti electrode was modified by placing the electrode in a 3% v/v APTES solution in ethanol for 3 hours under mild shaking. Next, the electrode was washed with ethanol 3 times (10 min incubation each time) and finally rinsed with distilled water. The electrode was then placed in a solution containing glutaraldehyde (1.5% v/v in H_2_O) for 1 hour. The electrode was further rinsed with HEPES buffer (10 mM, pH 7.2), and incubated with apoferritin (0.5 mg mL^−1^) for 1 hour. Afterward, the modified electrode was rinsed with HEPES buffer (10 mM, pH 7.2). Also in this case the pH change was triggered by applying −0.8 V *vs.* Ag/AgCl_sat_ for 90 minutes.

#### 4.3.4. Bioelectrochemical synthesis of CdSe NPs with apoferritin

The reaction mixture solution of 1 mM cadmium acetate and 5 mM selenourea was prepared in NH_3_/acetate buffer (3 mM, pH 6.5). The pH was changed by applying 0 V *vs.* Ag/AgCl, which caused oxygen reduction reaction (ORR) occurring at the electrode surface (potential applied for 90 minutes).

CdSe NPs can be synthesized in the absence of apoferritin but their size will not be controlled, resulting in NPs with high dispersion distribution in terms of size. To control the size of NPs and decrease the overpotential applied for ORR, apoferritin and laccase were immobilized on the surface of titanium electrode. The Ti electrode was modified by placing the electrode in a 3% v/v APTES solution in ethanol for 3 hours under mild shaking. Next, the electrode was washed with ethanol 3 times (10 min incubation each time) and finally rinsed with distilled water. The electrode was then placed in a solution containing glutaraldehyde (1.5% v/v in H_2_O) for 1 hour. The electrode was further rinsed with HEPES buffer (10 mM, pH 7.2), and incubated with laccase (3.6 mg mL^−1^) and apoferritin (0.5 mg mL^−1^) for 1 hour. Afterward, the modified electrode was rinsed with HEPES buffer (10 mM, pH 7.2). Also, in this case the pH change was triggered by applying 0 V *vs.* Ag/AgCl_sat_ for 90 minutes.

## Author contributions

P. B. and R. G. conceived the project and wrote the manuscript. A. T., B. A. and V. M. directly performed the measurements. L. T., P. B. and R. G. supervised A. T., B. A. and V. M. during the project. L. T., P. B. and R. G. are responsible for funding acquisition.

## Conflicts of interest

There are no conflicts to declare.

## Supplementary Material

NA-006-D3NA01046E-s001
