# Supplementary material for: Bioelectrochemically triggered apoferritin-based bionanoreactors: synthesis of CdSe nanoparticles and monitoring with leaky waveguides
Source: Nanoscale Adv. 2023 Dec 14;6(2):516–23. doi: 10.1039/d3na01046e (PMC10790968; doi:10.1039/d3na01046e)
Supplement: NA-006-D3NA01046E-s001 [file NA-006-D3NA01046E-s001.pdf]

Electronic Supplementary Material (ESI) for Nanoscale Advances.
This journal is © The Royal Society of Chemistry 2023

Electronic Supplementary Information

Bioelectrochemically Triggered Apoferritin-based Bionanoreactors: Synthesis of CdSe Nanoparticles and Monitoring with Leaky Waveguides

Angelo Tricase,[a,b,†] Bushra Alhenaki, [c,†] Verdiana Marchianò,[a,b] Luisa Torsi,[a,b] Ruchi Gupta,[c,*] and Paolo Bollella[a,b,*].

[a]Department of Chemistry, University of Bari Aldo Moro, Via E. Orabona, 4 – 70125 Bari, Italy

[b]Centre for Colloid and Surface Science, University of Bari Aldo Moro, Via E. Orabona, 4 – 70125 Bari, Italy

[c]School of Chemistry, University of Birmingham, Birmingham, B15 2TT, UK

CFM calibration curve S2
Kinetics experiments on electrochemical synthesis of CdSe NPs with apoferritin S3
UV-Vis calibration curve with pristine CdSe NPs S3
Fabrication of leaky waveguide (LW) devices S4
Procedure for analysis of 2D reflectivity curves of LWs S4
References S5

CFM calibration curve

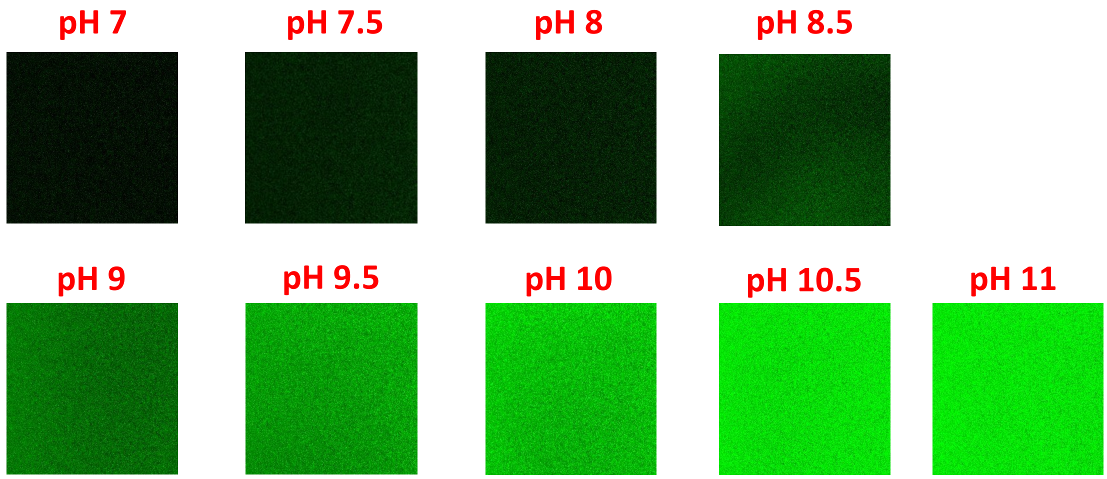

Figure S1. *Confocal microscope images recorded in a solution containing 10 µM FAM345 with different bulk pHs: 3 mM NH_3/acetate buffer + 100 mM Na_2SO_4 (pH varying from 7 to 11 every half pH unit). CFM images were recorded at 470-600 nm exciting at 405 nm.*

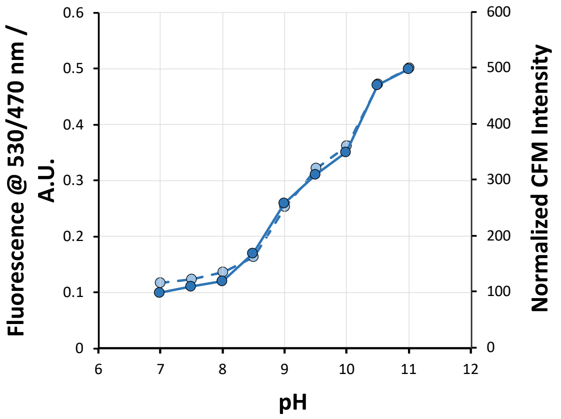

Figure S2. *Titration curves matching for 10 µM FAM345 measured with fluorometer (solid line) and confocal fluorescent microscope (CFM, dashed line). Left axis fluorescence in normalized arbitrary units and right axis CFM in normalized CFM intensity.*

Kinetics experiments on electrochemical synthesis of CdSe NPs with apoferritin

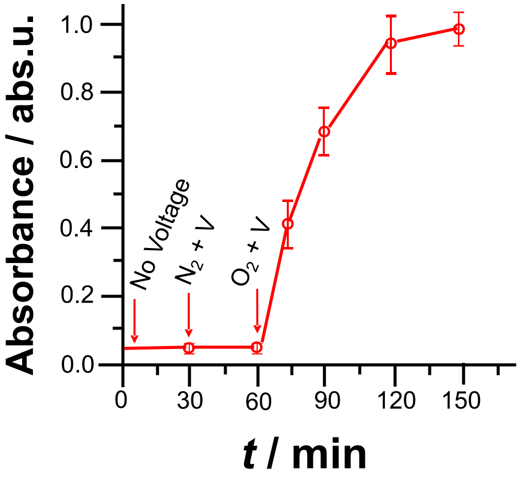

Figure S3. *UV-Vis kinetics measurements of reaction media after applying -0.8 V vs. Ag/AgCl for CdSe NPs synthesis upon local pH change induced by ORR at Ti electrodes.*

UV-Vis calibration curve with pristine CdSe NPs

Pristine CdSe NPs were characterized by recording UV-Vis spectra at different concentrations ranging between 0 and 2.5 mg/mL, as shown in Figure S4A. CdSe NPs in DI water showed a UV-Vis peak at 552 nm, which agrees with the results reported in the literature. [1] A calibration curve with the following equation, namely y=0.378*x* + 0.007 (n=3, R=0.995), was obtained (Figure S4B). This equation was used to estimate the concentration of CdSe obtained through all synthetic pathways.

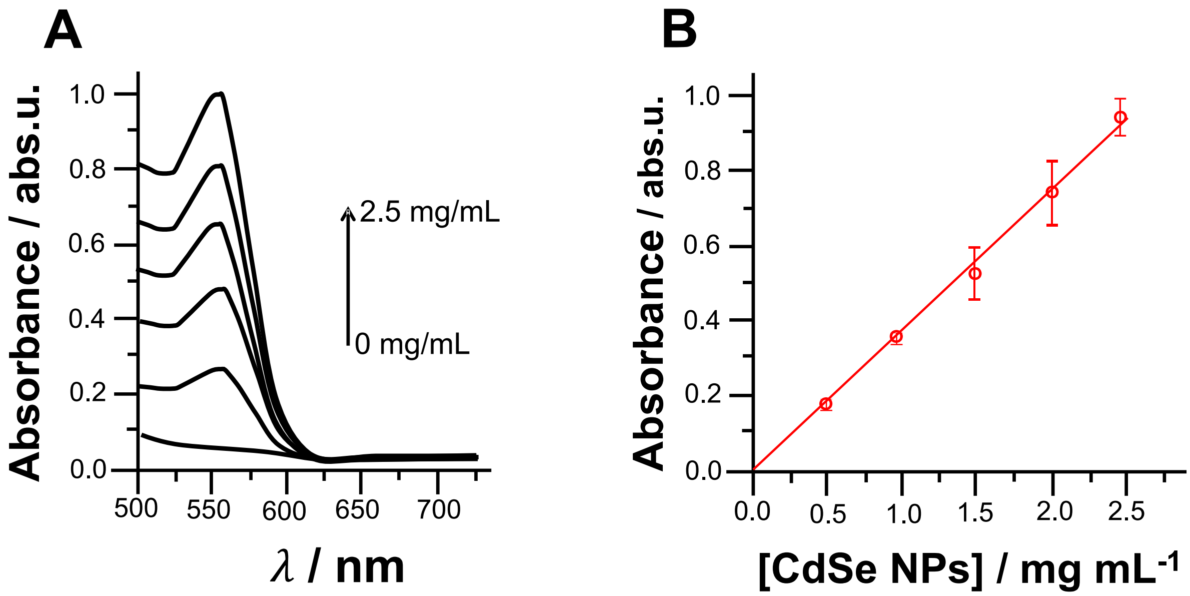

Figure S4. ***(A)*** *UV-Vis spectra of pristine CdSe NPs at different concentrations (0-2.5 mg/mL) in 3 mM NH_3/acetate buffer pH 6.5 + 100 mM Na_2SO_4;* ***(B)*** *calibration curve obtained from UV-Vis spectra of CdSe NPs.*

Fabrication of Leaky Waveguide (LW) devices

Microscope slides were cut into squares of ~25 × 25 mm^2. Glass squares were cleaned in decon 90 solution, de-ionised water, and ethanol by sonication for 30 min each. Chitosan was purified using the protocol described previously [2]. The purified chitosan was dissolved in 0.1 M acetic acid under continuous stirring for 18 h to form a 1% (w/v) solution. 200 µL of chitosan solution was spin coated onto the glass substrate at a spin speed of 900 rpm for 30 s with acceleration of 100 rpm/s inside a laminar flow cabinet. The spin coated substrates were placed inside an incubator maintained at 25 °C and humidity of 75–80% for 3 min. The chitosan films were then crosslinked by immersing them in 0.03% (v/v) GA prepared in 10 mM HEPES buffer, pH 7.4 for 5 min, washed with HEPES buffer to remove unreacted GA, and stored in HEPES buffer until further use.

Procedure for analysis of 2D reflectivity curves of LWs

Wavelength calibration of the instrumentation was carried out by inserting interference filters of known peak wavelength between the grating and camera before taking an image. Intensity profiles were extracted from these images and a Gaussian fit was performed to determine the pixel position for each filter. A linear fit to these pixel positions then permitted the wavelength corresponding to any pixel position to be determined. Subsequently, images from the LW devices were analysed using an ImageJ macro written for the purpose. A region of interest was defined by straight lines bracketing the resonance position as shown in Figure S5(A). The resonance profile was extracted in this region of interest in rectangular areas 24 pixels wide, resulting in 229 profiles (image width of 5496 pixels divided by 24). For each profile, background correction was applied by taking a straight line between the start and end of the profile and subtracting the profile values from the values given by this line. This resulted in a peak rather than a dip, from which the maximum value was extracted. The resulting 229 maxima were extracted from each image in turn and the resulting data exported to Sigmaplot for further processing and graphing. Intensity values were extracted from the white light plot in Figure S5(B) and used as the intensity reference. A Sigmaplot macro was used to take the maxima and calculate the absorbance based on the profile maxima and the white light intensities at each of the 229 wavelength values. This process was applied to each set of images to obtain time resolved LW spectra of the chemically and bioelectrochemically formed NPs

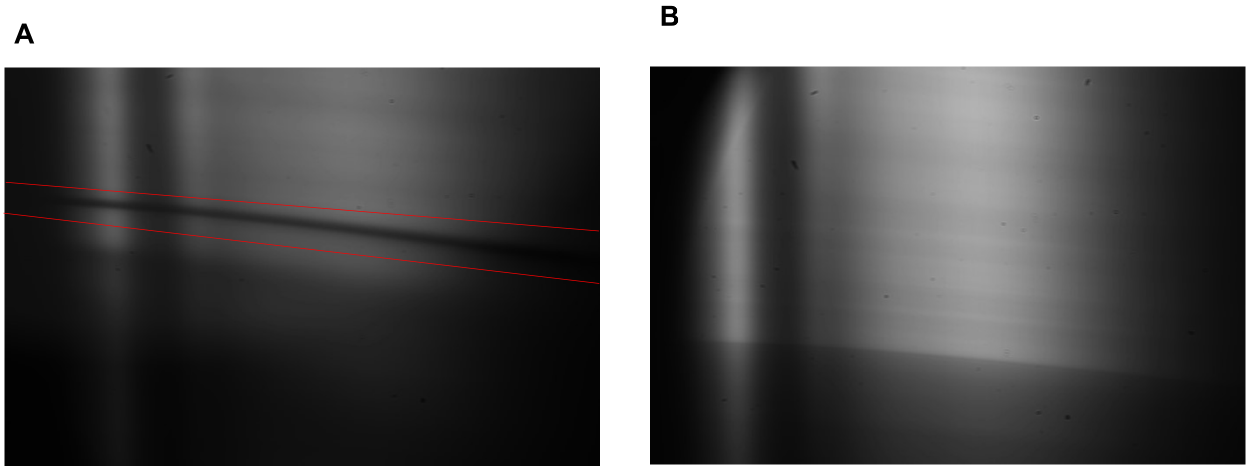

Figure S5. ***(A)*** *Sample image showing the region taken for analysis of the depth of the resonance (I),* ***(B)*** *White light image of a glass slide with no LW to act as the intensity reference (I_0)*

References

[1] H. S. Mansur, A. A. Mansur, Mater. Chem. Phys. 2011, 125(3), 709-717.
[2] R. Signini, S.P. Campana Filho, Polymer Bulletin 1999, 42, 159-166.
